# Assessing the resilience of a key health service: The response of acute surgical provision in England to the disruption of the COVID-19 pandemic

**DOI:** 10.1177/13558196261426970

**Published:** 2026-03-05

**Authors:** Andrew Hutchings, Orlagh Carroll, Geoff Bellingan, David Cromwell, S Ramani Moonesinghe, Susan J Moug, Neil Smart, Ravinder Vohra, Robert J Hinchliffe, Richard Grieve

**Affiliations:** 1 Department of Health Services Research and Policy, 4906 London School of Hygiene & Tropical Medicine, London, UK; 2 Bloomsbury Institute for Intensive Care Medicine, Division of Medicine, 4919 University College London, London, UK; 3 Department of Targeted Intervention, Division of Surgery and Interventional Science, 4919 University College London, London, UK; 4 Department of Surgery, Royal Alexandra Hospital, NHS Greater Glasgow and Clyde, Paisley, UK; 5 159028 College of Medicine and Health, Royal Devon and Exeter Hospital, Royal Devon University Healthcare NHS Foundation Trust, Exeter, UK; 6 City Hospital, 9820 Nottingham University Hospitals NHS Trust, Nottingham, UK; 7 Bristol Surgical Trials Centre, University of Bristol, Bristol, UK

**Keywords:** health system resilience, COVID-19, emergency surgery

## Abstract

**Objective:**

International health systems had the opportunity to assess the resilience of core health services to severe disruption following the onset of the COVID-19 pandemic. This paper examines the resilience of a core health service to shocks from COVID-19. We compare outcomes following emergency admissions in England during the second (Winter 2020/21) and third (Winter 2021/22) major waves of COVID-19 with the first wave and historic admissions from 2016 to 2019.

**Methods:**

This cohort study included adult emergency admissions for five common acute surgical conditions: appendicitis, symptomatic gallstone disease, intestinal obstruction, symptomatic diverticular disease, and abdominal wall hernia in 122 acute hospital Trusts in England. Participants were 647,367 admissions in the hospital episode statistics (HES) inpatient database including 34,560 in the second wave and 36,628 in the third wave. Outcome was all-cause mortality at 90 days.

**Results:**

There were 1308 deaths in wave two (3.8% of admissions) and 1235 (3.4%) in wave three compared with 3431 (3.4%) in the historic cohort and 577 (4.7%) in wave one. Compared with pre-COVID admissions, we found weak evidence of increased mortality in the second wave. There was no evidence of increased mortality in the third wave, compared to historic cohorts the case-mix adjusted odds ratios were: appendicitis 0.96 (95% CI 0.49–1.87); gallstone disease 1.27 (95% CI 0.94–1.72); diverticular disease 1.04 (95% CI 0.79–1.36); hernia 1.06 (95% CI 0.76–1.47); and intestinal obstruction 1.02 (95% CI 0.87–1.19).

**Conclusions:**

By the end of wave three, outcomes for emergency admissions with five common acute conditions had returned to pre-pandemic levels. Lessons learnt during the disruption of the first wave of COVID-19 helped the NHS in England adapt emergency surgical services during subsequent waves. These findings emphasise the importance of maintaining, or quickly restoring core service capacity to help patient outcomes return to pre-pandemic levels.

## Introduction

National health systems will need to be resilient to future pandemics. Resilience can be defined as the system’s ability to absorb, recover from and adapt to disruptions by learning lessons from previous shocks.^1–4^ The multiple waves of the COVID-19 pandemic presented different challenges and led to medium-term disruption to health services. Evidence about how health service provision adapted across these different waves of COVID-19 can inform plans for future pandemics and other crises.^5^

Although the COVID-19 pandemic period offered opportunities for studying health system resilience, there is a lack of international evidence about how health systems adapted across the time course of the pandemic. Studies that have compared service volumes across different health systems have lacked the information on the characteristics of the populations, the health services provided, patient outcomes and how each of these changed over the time course of the pandemic.^1,6–8^ Previous studies have assessed the impact of the early waves of the pandemic, but have not considered the subsequent health service response.^1,6–8^ The evidence base available to policy-makers about the resilience of health services to medium-term disruption is therefore limited.

Acute surgery is an essential health service that covers a broad range of conditions and population groups. During the first wave of COVID-19, many countries redeployed ventilators, hospital space and personnel to try and mitigate the substantial disruption to acute surgery provision. Despite these mitigation strategies, studies observed substantial reductions in emergency admissions, rates of emergency surgery, critical care use and durations of hospital stay during the first wave of the pandemic.^9^ For common acute surgical conditions, the first wave of COVID-19 in England was associated with a sharp drop in emergency admissions and higher 90-days mortality.^9^ During the second major wave of infection from the alpha variant, the increase in testing,^10^ and the vaccine rollout which started in December 2020 meant that the risks of adverse outcomes following COVID-19 infection were reduced, and there was less staff absence from NHS hospitals.^11^ Hospitals introduced triage systems and infection control measures, with less displacement of ventilators, clinicians and ICU beds to care for patients with confirmed COVID-19 infection.^12^ Updated guidelines were issued reflecting new evidence on best practice for emergency surgery for patients presenting with common acute conditions.^13^ In particular, initial concerns about laparoscopic surgery during COVID-19,^14^ were shown to be false, and previous thresholds for emergency surgery were reinstated. The emergence of the omicron variant and the third major wave of COVID-19 in England occurred after the initial vaccine rollout and booster and recorded infections exceeded previous waves, peaking in early January 2022, with higher levels of NHS staff absence than the second wave.^15^

While policy-makers attempted to learn lessons and adapt acute surgical provision following the first wave of the pandemic, there is little evidence to assess the overall impact on health of the mitigation strategies taken. This crucial gap in evidence hinders any attempt to ‘learn lessons’ that could help health systems maintain acute care when faced with a future crisis.

This paper aimed to address two related questions pertaining to health service resilience. First, was all-cause mortality following emergency admission for common acute conditions during the second and third waves of COVID-19 in England higher than in a historic cohort of corresponding admissions? Second, was any relative increase in all-cause mortality during these waves smaller than the relative increase in the first wave? We considered the following acute surgical conditions: acute appendicitis, acute symptomatic gallstone disease, intestinal obstruction (small or large bowel), symptomatic diverticular disease, and abdominal wall hernia.

## Methods

### Study population

The study used Hospital Episodes Statistics (HES) data for England to define emergency admissions to 122 NHS acute hospital Trusts for five acute surgical conditions.^22^ The definitions of the study population and of emergency surgery have been reported previously as part of the ESORT study,^16^ and the assessment of the first wave of COVID-19.^9^ In brief, patients aged 18 years or more were eligible if a finished consultant episode met the following criteria: (i) included a main diagnosis with an International Classification of Diseases, tenth revision (ICD-10) diagnosis code specified by consensus of a clinical panel, (ii) was within an emergency admission through the Emergency Department, or from a primary care referral; (iii) was under a consultant general or sub-specialty surgeon, or a surgeon working in the general surgery specialty; and (iv) was the first or second episode within the admission. For intestinal obstruction, a relevant diagnosis could appear in the second diagnosis field if the main diagnosis was colorectal cancer. For all five conditions exclusion criteria were: incomplete discharge information; a prior emergency admission with a relevant diagnosis in the previous 12 months or an eligible emergency surgery procedure in the previous 3 months.

### Study periods

For this study the period for eligibility was 1 April 2016 to 30 June 2022. We included 122 acute general NHS Trusts in existence on 31 March 2022. The second wave of COVID-19 in England (alpha variant dominant) was defined as the period from 4 November 2020 to 11 March 2021 (week 45, 2020 to week 10, 2021) and ran from the start of the second national lockdown to the reopening of schools.^17^ The third wave (omicron variant dominant) was defined as the equivalent period in 2021/22, as in both waves infections peaked at the beginning of January.^17^ For waves two and three the historic comparator was defined by the same calendar period in each of the 3 years from 2016/17 to 2018/19. Wave one was defined as weeks 11 to 19 in 2020 [6] (11th March to 12 May 2020) with a historic comparison of the same period in the years 2017–2019.^17^ We choose to define the historic comparator across 3 years as annual excess winter deaths can be highly variable.

### Patient-level covariates

Patient characteristics extracted from HES data were: age (years), sex, ethnicity, Index of Multiple Deprivation (IMD), diagnostic subcategories and COVID-19 infection. We derived Charlson comorbidity index,^18^ and secondary care administrative records frailty (SCARF) index,^19^ scores using HES data. The SCARF frailty index was developed and validated in a surgical cohort. It defines patients as being ‘fit’ or having mild, moderate or severe frailty based on the accumulation of deficits that cover functional impairment, geriatric syndromes, problems with nutrition, cognition and mood and medical comorbidities. COVID-19 infection was defined if there were ICD-10 diagnoses codes recorded for COVID-19 with or without laboratory confirmation in the index episode, or if either of these codes or a history of COVID or a post-COVID condition was reported in any subsequent episode up to 90 days.

### Definition of emergency surgery

We defined emergency surgery from a list of Office of Population Censuses and Surveys (OPCS) procedure codes selected by a clinical panel provided the surgery was within a time window of 3 days (hernia), 7 days (appendicitis, gallstone disease, intestinal obstruction), or any time within the emergency admission (diverticular disease).^16^ Those admissions that met the eligibility criteria but who did not have the required surgical procedures within the respective time windows were included but designated as having had ‘no emergency surgery’. The patients in the ‘no emergency surgery’ group may have had either: medical management with no surgical procedures; surgical procedures that were not classified as relevant for the condition in question, or surgical procedures that were relevant for the condition, but were received after the designated time window.

### Outcomes

The outcome measure was all-cause mortality at 90 days from the index episode using date of death from linkage to the Office for National Statistics (ONS) death records. Complete follow-up data were available for all patients.

### Statistical analysis

We estimated the association of emergency admission during the second and third waves of COVID-19 with 90-days all-cause mortality using multiple logistic regression adjusting for case mix, seasonality and temporal trends. Case mix variables were age, sex, ethnicity, diagnostic subcategories, Index of Multiple Deprivation (IMD) quintiles, number of Charlson comorbidities, and Secondary Care Administrative Records Frailty (SCARF) frailty index. The functional form of age was assessed using the fractional polynomial selection algorithm.^20^ NHS Trusts were included as random intercepts and teaching hospital status as a fixed effect. Missing data were present for sex (<0.01% missing) and IMD quintiles (1.3% missing). We applied complete case analysis.

The seasonality covariate was defined by four levels: January-March, April-June, July-September and October-December. To allow for both seasonality and temporal trends, the analysis model included an interaction between the year of admission and the seasonality covariate. Year was included as a continuous variable and assumed to follow a linear trend with respect to mortality.

We reported adjusted odds ratios (ORs) of mortality following each wave versus the historic comparator. The relative impact of waves two and three versus wave one was summarised by the ratio of adjusted ORs, overall and by whether or not the patient had emergency surgery, The model was expanded to include a three-way interaction between emergency surgery or not, period, and COVID wave. We reported the estimated ORs from these two- and three-way interactions in forest plots that summarised the impact of (a) waves two and three compared to their historic comparators, (b) wave two and three versus wave one, and (c) wave one compared to its historic comparator.

In sensitivity analyses, we excluded patients with recorded evidence of COVID infection in the index episode or up to 90 days; adjusted for seasonality using the day of admission rather than the 3-month period, or by including an interaction between the month and year of admission; and defined the outcome as 30- rather than 90- day mortality.

The design and interpretation of this study was informed by a patient and public advisory group at online workshops held in July 2020 September 2021, and May 2022. Further details are available on the ESORT study website (https://www.lshtm.ac.uk/research/centres-projects-groups/esort).

## Results

### Overall cohort

**Figure 1.**
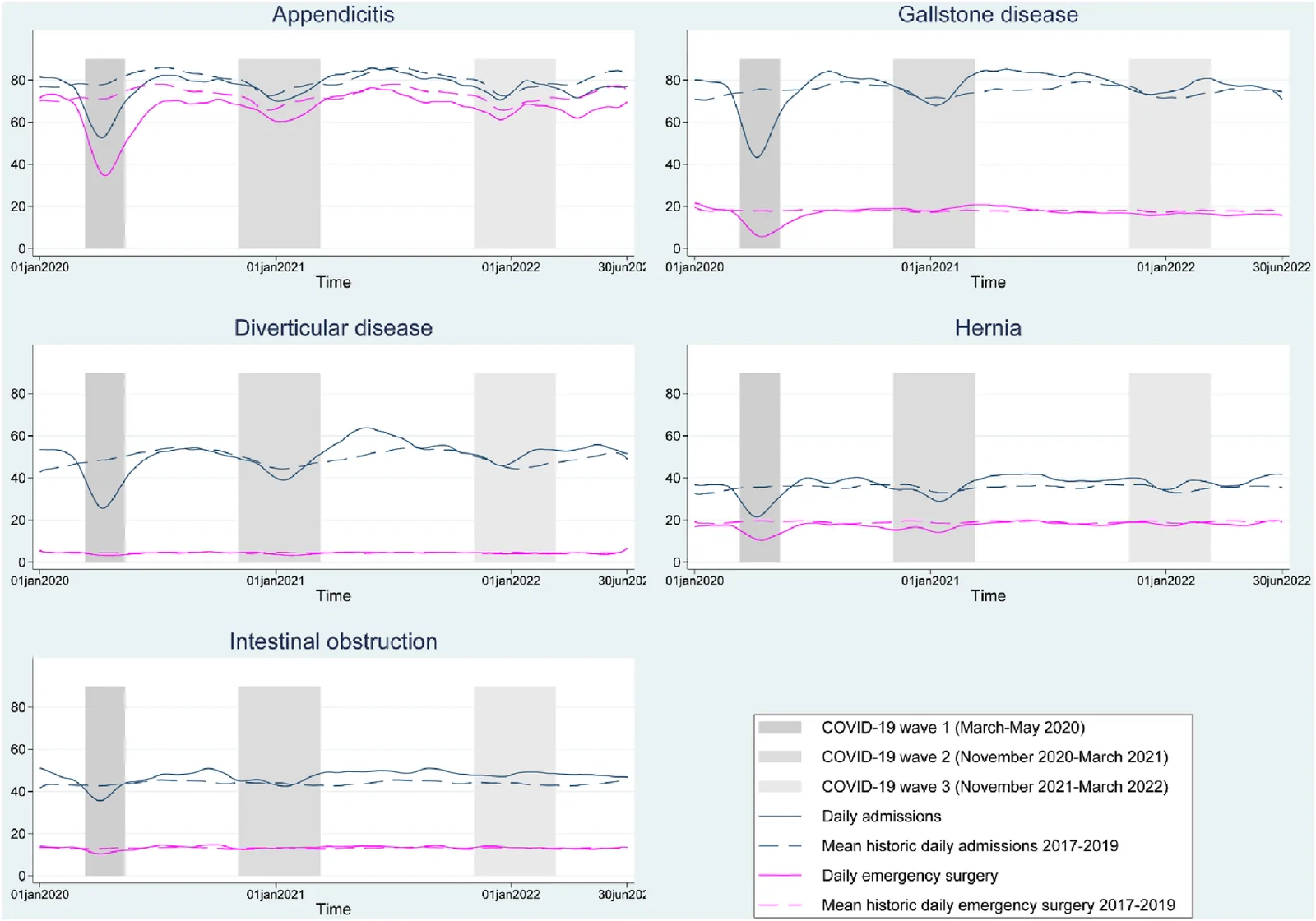
Mean daily admissions and emergency surgery numbers (smoothed) for five acute conditions from January 2020 to June 2022 compared with historic averages.

We included 647,367 patients admitted in wave one (*n* = 12,403), two (*n* = 34,560) and three (*n* = 36,628) respectively (see exclusions in Supplemental Tables 1–5). The reductions in the numbers of emergency admissions and in the proportions who had emergency surgery were less in waves two and three than in the first wave, and the weekly numbers of admissions were similar to corresponding periods for the historic comparators (Figure 1) (Table 1). Compared to the historic comparators, patients admitted in waves two and three were more likely to have moderate or severe frailty but were otherwise similar. The large reduction in laparoscopic procedures in wave one was not seen in waves two and three. Table 2 reports all-cause mortality at 90-days before any casemix adjustment by COVID-19 wave and comparator period.

**Table 1.** Patient characteristics of emergency admissions for five acute conditions, by COVID-19 wave and comparator period.

	Numbers		Case mix				Emergency surgery		
	Number: *n*	Mean admissions per week	Mean age (sd)	Sex (female): *n* (%)	Charlson comorbidity (>1): *n* (%)	SCARF frailty (moderate or severe): *n* (%)	Number (%)	Laparoscopic surgery: *n* (%)	COVID diagnosis from admission to 90 days: *n* (%)
Appendicitis									
Wave 1	3636	404.0	41.3 (17.3)	1661 (45.7)	99 (2.7)	182 (5.0)	2538 (69.8)	1445 (56.9)	90 (2.5)
Wave 1 comparator	14,846	549.9	40.2 (17.2)	6958 (46.9)	381 (2.6)	558 (3.8)	13,533 (91.2)	11,613 (85.8)	n/a
Wave 2	9447	516.6	41.2 (17.2)	4346 (46.0)	291 (3.1)	527 (5.6)	8158 (86.4)	7384 (90.5)	324 (3.4)
Wave 3	9633	531.0	42.1 (17.6)	4590 (47.6)	347 (3.6)	631 (6.6)	8336 (86.5)	7599 (91.2)	419 (4.4)
Wave 2/3 comparator	28,870	529.0	39.8 (17.0)	13,263 (45.9)	785 (2.7)	1098 (3.8)	26,340 (91.2)	22,242 (84.4)	n/a
Gallstone disease									
Wave 1	3049	338.8	56.0 (18.6)	1955 (64.1)	340 (11.2)	500 (16.4)	463 (15.2)	286 (61.8)	103 (3.4)
Wave 1 comparator	14,195	525.7	55.8 (19.0)	9493 (66.9)	1601 (11.3)	2078 (14.6)	3387 (23.9)	2931 (86.5)	n/a
Wave 2	9446	516.6	56.0 (18.5)	6135 (64.9)	1143 (12.1)	1745 (18.5)	2421 (25.6)	2054 (84.8)	503 (5.3)
Wave 3	9673	533.2	57.1 (18.6)	6265 (64.8)	1247 (12.9)	1876 (19.4)	2083 (21.5)	1722 (82.7)	525 (5.4)
Wave 2/3 comparator	27,261	499.5	55.4 (18.9)	17,992 (66.0)	2900 (10.6)	3729 (13.7)	6756 (24.8)	5858 (86.7)	n/a
Diverticular disease									
Wave 1	1835	203.9	63.0 (15.7)	1024 (55.8)	223 (12.2)	386 (21.0)	208 (11.3)	14 (6.7)	89 (4.9)
Wave 1 comparator	9171	339.7	63.0 (15.5)	5405 (58.9)	1164 (12.7)	1645 (17.9)	827 (9.0)	104 (12.6)	n/a
Wave 2	5694	311.4	62.2 (15.7)	3336 (58.6)	708 (12.4)	1285 (22.6)	512 (9.0)	61 (11.9)	314 (5.5)
Wave 3	6384	351.9	62.3 (15.7)	3734 (58.5)	829 (13.0)	1396 (21.9)	530 (8.3)	66 (12.5)	306 (4.8)
Wave 2/3 comparator	17,101	313.4	63.5 (15.6)	10,043 (58.7)	2193 (12.8)	3128 (18.3)	1683 (9.8)	234 (13.9)	n/a
Hernia									
Wave 1	1499	166.6	64.1 (17.7)	526 (35.1)	202 (13.5)	314 (20.9)	713 (47.6)	20 (2.8)	60 (4.0)
Wave 1 comparator	6759	250.3	62.8 (18.4)	2523 (37.3)	836 (12.4)	1311 (19.4)	3669 (54.3)	133 (3.6)	n/a
Wave 2	4228	231.2	63.3 (18.0)	1425 (33.7)	520 (12.3)	985 (23.3)	2046 (48.4)	87 (4.3)	170 (4.0)
Wave 3	4789	264.0	63.4 (18.0)	1665 (34.8)	632 (13.2)	1147 (24.0)	2340 (48.9)	106 (4.5)	230 (4.8)
Wave 2/3 comparator	12,802	234.6	62.7 (18.5)	4614 (36.0)	1545 (12.1)	2446 (19.1)	7146 (55.8)	278 (3.9)	n/a
Intestinal obstruction									
Wave 1	2384	264.9	68.0 (16.6)	1279 (53.6)	449 (18.8)	739 (31.0)	692 (29.0)	44 (6.4)	115 (4.8)
Wave 1 comparator	8107	300.3	67.8 (16.6)	4346 (53.6)	1445 (17.8)	2130 (26.3)	2446 (30.2)	200 (8.2)	n/a
Wave 2	5745	314.2	67.7 (16.9)	3139 (54.6)	1066 (18.6)	1892 (32.9)	1667 (29.0)	203 (12.2)	471 (8.2)
Wave 3	6149	338.9	67.9 (16.6)	3186 (51.8)	1209 (19.7)	2064 (33.6)	1716 (27.9)	198 (11.5)	423 (6.9)
Wave 2/3 comparator	16,294	298.6	67.3 (17.0)	8565 (52.6)	2870 (17.6)	4122 (25.3)	4943 (30.3)	363 (7.3)	n/a

**Figure 2.**
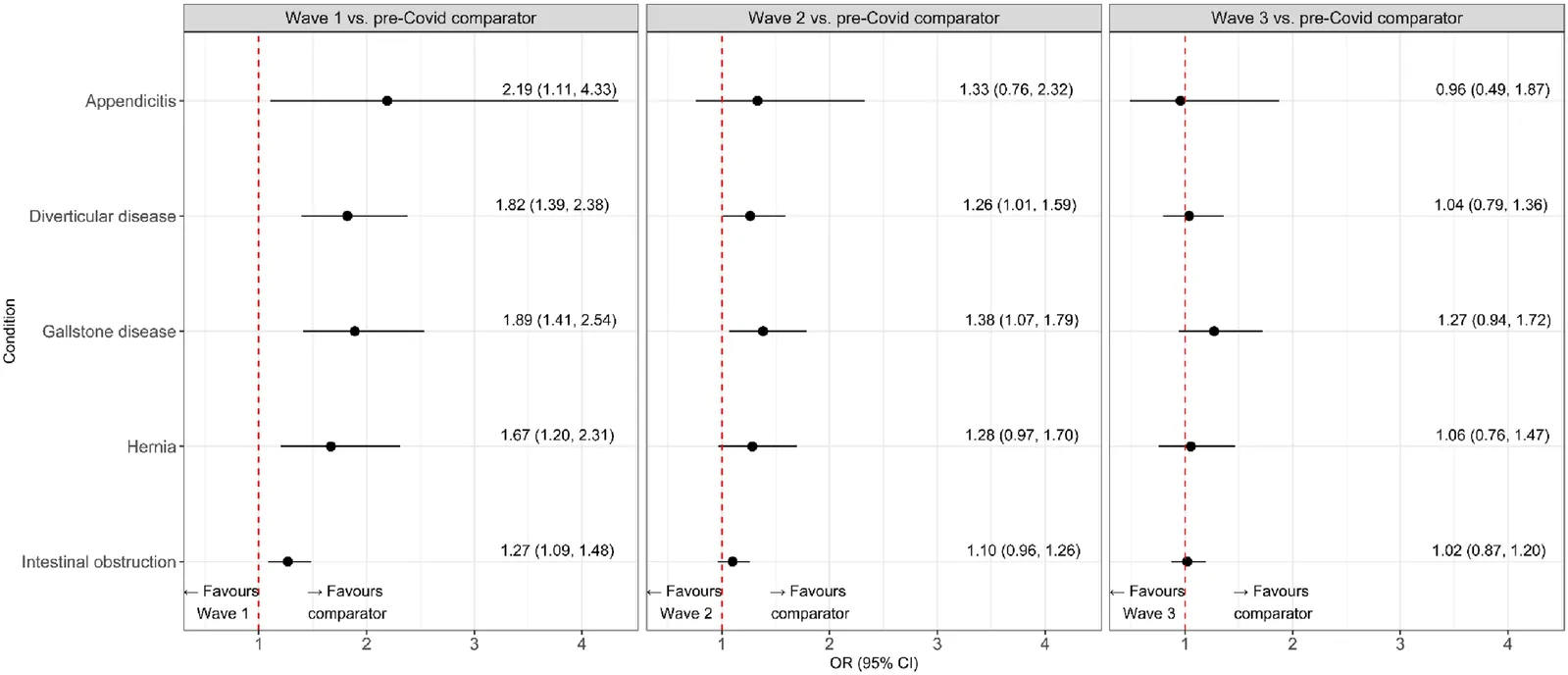
Forest plots of impact of COVID-19 Waves 1–3 versus historic comparators on 90-days all cause mortality in five acute conditions. Results are reported as estimated odds ratios (ORs) with 95% confidence intervals (CI).

There was weak evidence of increased case-mix adjusted mortality during wave two versus the historical comparator periods for gallstone disease and diverticular disease, but not for the other three conditions (Figure 2). There was a lack of evidence for increased mortality in wave three versus historical comparators with adjusted ORs for gallstone disease of 1.27 (95% CI 0.94–1.72) and between 0.96 and 1.06 for the other four conditions (Figure 2). For all five conditions, case-mix adjusted mortality was higher for admissions during wave one versus historic comparators.

Direct comparison of the impact of wave three versus wave one provided evidence of reduced impact on mortality for gallstone disease (OR 0.57, 95% CI 0.40–0.81), diverticular disease (OR 0.67, 95% CI 0.45–0.996), hernia (OR 0.63, 95% CI 0.41–0.98) and intestinal obstruction (OR 0.81, 95% CI 0.65–0.99). The largest reduction in mortality was for appendicitis (OR 0.44, 95% CI 0.18–1.07) (Supplemental Figure 1).

**Figure 3.**
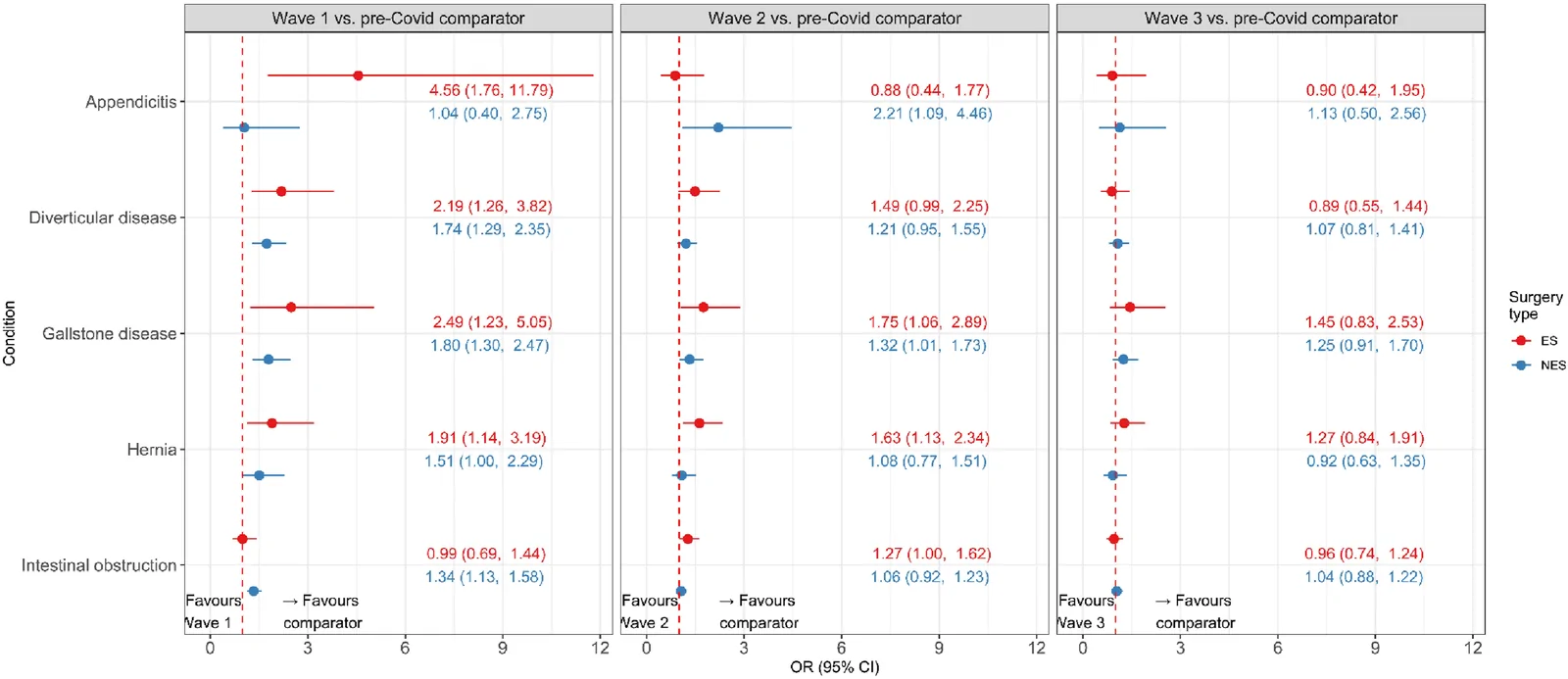
Forest plots of impact of COVID-19 Waves 1–3 versus historic comparators on 90-days all cause mortality in five acute conditions, according to whether patients had emergency surgery (ES) or alternative ‘no emergency surgery (NES)’ strategies. Results are reported as estimated odds ratios (ORs) with 95% confidence intervals (CI).

For patients who had emergency surgery, case-mix adjusted mortality was higher during wave two for gallstone disease and hernia (Figure 3), but in wave three there was a lack of evidence for increased mortality for all conditions (Figure 3).

For patients who did not have emergency surgery there was evidence of increased mortality during wave two in appendicitis and gallstone disease, but not for the other conditions. In wave three there was no evidence for increased mortality.

Sensitivity analyses excluding patients with COVID-19 recorded in the index episode or at any time to 90 days resulted in similar or smaller ORs for case-mix adjusted mortality in waves two and three versus their historic comparators (Supplemental Figures 2 and 3). None of the sensitivity analyses reported increased mortality in wave three versus the historic comparator.

**Table 2.** All-cause mortality at 90-days overall and by receipt of emergency surgery, by COVID-19 wave and comparator period.

	Overall		Emergency surgery		No emergency surgery	
	*n*	Deaths within 90 days: *n* (%)	*n*	Deaths within 90 days: *n* (%)	*n*	Deaths within 90 days: *n* (%)
Appendicitis						
Wave 1	3636	16 (0.44)	2538	9 (0.35)	1098	7 (0.64)
Wave 1 comparator	14,846	26 (0.18)	13,533	10 (0.07)	1313	16 (1.22)
Wave 2	9447	39 (0.41)	8158	15 (0.18)	1289	24 (1.86)
Wave 3	9633	35 (0.36)	8336	17 (0.20)	1297	18 (1.39)
Wave 2/3 comparator	28,870	94 (0.33)	26,340	61 (0.23)	2530	33 (1.30)
Gallstone disease						
Wave 1	3049	73 (2.39)	463	13 (2.81)	2586	60 (2.32)
Wave 1 comparator	14,195	200 (1.41)	3387	27 (0.80)	10,808	173 (1.60)
Wave 2	9446	167 (1.77)	2421	29 (1.20)	7025	138 (1.96)
Wave 3	9673	162 (1.67)	2083	23 (1.10)	7590	139 (1.83)
Wave 2/3 comparator	27,261	413 (1.51)	6756	53 (0.78)	20,505	360 (1.76)
Diverticular disease						
Wave 1	1835	105 (5.72)	208	27 (13.0)	1627	78 (4.79)
Wave 1 comparator	9171	298 (3.25)	827	56 (6.77)	8344	242 (2.90)
Wave 2	5694	237 (4.16)	512	51 (9.96)	5182	186 (3.59)
Wave 3	6384	210 (3.29)	530	31 (5.85)	5854	179 (3.06)
Wave 2/3 comparator	17,101	609 (3.56)	1683	120 (7.13)	15,418	489 (3.17)
Hernia						
Wave 1	1499	63 (4.20)	713	22 (3.09)	786	41 (5.22)
Wave 1 comparator	6759	186 (2.75)	3669	70 (1.91)	3090	116 (3.75)
Wave 2	4228	152 (3.60)	2046	63 (3.08)	2182	89 (4.08)
Wave 3	4789	137 (2.86)	2340	57 (2.44)	2449	80 (3.27)
Wave 2/3 comparator	12,802	406 (3.17)	7157	174 (2.43)	5656	232(4.10)
Intestinal obstruction						
Wave 1	2384	320 (13.4)	692	42 (6.07)	1692	278 (16.4)
Wave 1 comparator	8107	914 (11.3)	2446	162 (6.62)	5661	752 (13.3)
Wave 2	5745	713 (12.4)	1667	131 (7.86)	4078	582 (14.3)
Wave 3	6149	691 (11.2)	1716	116 (6.76)	4433	575 (13.0)
Wave 2/3 comparator	16,294	1909 (11.8)	4943	339 (6.86)	11,351	1570 (13.8)

## Discussion

Lessons learnt during the disruption of the first wave of COVID-19 helped the NHS in England adapt the provision of emergency surgery during subsequent waves. By the end of wave three numbers of emergency admissions, rates of surgery and outcomes for people with five common acute conditions had returned to pre-pandemic levels. The recovery in outcomes was similar irrespective of whether or not people had emergency surgery.

This paper offers new insights about how a crucial health service, acute surgical provision in England, was resilient to the COVID-19 pandemic. An international comparison found that the impact of the pandemic on surgical activity in the UK in 2020 was larger than in most other countries but also showed the greatest improvement in 2021.^6^ Zhong et al. found that in the USA the health system demonstrated an adaptive response, with 90% of the system performing better during the second wave versus the first.^1^ These studies did not consider the impact on patient outcomes, nor did they allow for differences in case-mix across the different waves of the pandemic, which could partly explain the apparent improvements following the initial shock. A multicentre study from France found evidence of increased hospital mortality in surgical patients in the first and second waves compared with pre-COVID periods but did not consider admissions after 2020.^21^

We report case-mix adjusted mortality for emergency admissions following five different surgical conditions across several waves of COVID-19 (2020–2022). We considered the ‘resilience’ of the health service according to a broad definition of resilience which deliberately included “learning from the past,” in this case, policy, management and professional learning from the first wave of the COVID-19 pandemic. Our study design allowed us to suggest three interlinked reasons as to how a crucial health service can adapt to a crisis and restore outcomes to pre-pandemic levels. These factors contributed to better preparedness for the second and third waves as hospitals and surgical teams adapted to the evolving pandemic.^22^

First, in waves two and three acute service provision benefited from the national response to the pandemic including the vaccination programme (rollout in wave two), widespread testing, and measures to reduce transmission such as lockdowns (waves one and two) and social distancing. Although the number of infections recorded peaked in wave three, these infections had less impact on NHS staff with average daily NHS staff absences of eight out of 100 employees at the peaks of waves two and three, versus 12 out of 100 employees in wave one making it easier to maintain hospital capacity.^15^ The clinical management of COVID in primary and secondary care also improved over the course of the pandemic.^23^ Despite higher overall hospital admissions, and pressure on critical care capacity in wave two,^24^ hospitals were better prepared for the later waves with improved systems for triage, infection control, staffing and management of critical care.^11,12^

Second, before the COVID-19 pandemic the NHS in England already had issued plans to prioritise emergency care over elective activity in the winter period. Following the outbreak of COVID-19 specific guidance was issued to surgeons on 20 March 2020 which identified the maintenance of emergency surgery capabilities as the first priority, and protecting and preserving the workforce as the second.^25^ The guidance highlighted that maintaining emergency surgery capacity could mean surgeons working ‘outside’ or ‘beyond individuals’ comfort zones’, and staff redeployment was widespread in the first wave as hospitals expanded critical care capacity to try and meet demand.^26^ The guidelines for emergency surgery reinstated laparoscopic surgery in waves two and three.^14^

Third, the direct effect of acquiring COVID-19 infection either during an emergency admission or after hospital discharge, could explain any excess mortality in waves 1–3 compared to pre-COVID periods. However, our sensitivity analyses found that excluding those who had a COVID-19 infection recorded, had little impact on the estimates of excess mortality.

Other studies have also identified factors that may explain health service resilience. Arsenault et al. found that most of the observed reduction in service provision was due to ‘supply-side’ rather than ‘demand-side’ factors.^7^ There is also some previous evidence that the more resilient health systems are those with spare capacity prior to the initial shock, particularly in the number of physicians available.^6,27^ Adaptation is also helped by physicians working collaboratively across settings, which in our study was exemplified by the mobility of staff who moved to critical care to help maintain ICU capacity which is essential for some patients following emergency surgery.

Previous research has suggested that for acute and emergency care a lack of spare capacity make these services especially vulnerable to disruption from shocks.^6^ In our setting, providers were able to maintain capacity by drawing on previous evidence and protocols to triage some patients to alternatives to emergency surgery, such as antibiotics for patients with acute appendicitis. Randomised controlled trials (RCTs) have compared medical management to emergency surgery for selected patients with uncomplicated acute appendicitis. These RCTs have reported equivocal results.^28,29^ Although the CODA trial reported similar 90-days outcomes between those randomised to receive antibiotics versus emergency surgery, almost one third of the antibiotics group subsequently had surgery.^28^ The COMMA trial reported improved Health-Related Quality of Life at 3 months in the appendectomy versus medical management group,^29^ and appendectomy remains the default choice for the majority of patients who present with uncomplicated acute appendicitis in the UK.^30^

### Limitations and research priorities

The large reductions in emergency admissions and increases in mortality rates in wave one may have led to differences in the case-mix of patients who presented in wave two or three versus historical comparison periods according to unmeasured variables. There is therefore a risk of residual confounding. A COVID-19 diagnosis was not included in the case-mix adjustment due to limited availability of testing in the first wave, and uncertainty in differentiating between COVID present at the start of an episode (a confounder), and that acquired during the episode (a mediator). The main outcome of interest was case-mix adjusted all-cause mortality, and information was not available on other measures of interest such as health-related quality of life. The study did not consider spillover effects, in particular on the backlog of people waiting for elective surgical procedures. The reduced capacity for elective surgery has led to medium-term disruption, and increased waiting times across many conditions including for gallstone disease, trauma and orthopedics, oral surgery, plastic surgery and cancer surgery. The percentage of patients diagnosed with cancer who do not have curative surgery within 1 month of the decision to operate increased during the pandemic and remains higher than national targets in many NHS Trusts. An urgent research priority is therefore to identify ways to reduce waiting times for elective procedures while also maintaining sufficient capacity for emergency surgery in anticipation of future crises. One option is to further invest in elective surgical hubs to help ringfence both elective and emergency surgical capacity for high volume procedures, for example for gall bladder disease or hernia repair.^31^

### Implications for policy

It is impossible to fully protect a health and care service from the consequences of crises such as the COVID-19 pandemic. However, this study suggests that for emergency admissions in the NHS acute surgical provision in England adapted during the second and third waves of the pandemic, following the disruption of the first wave. Examples of adaptations included: reverting to laparoscopic surgery as soon as evidence emerged in support of patient safety, transferring staff to maintain critical care capacity, providing alternatives to emergency surgery to maintain acute surgical capacity, and rapidly updating guidance through national networks.

## Conclusion

Our paper generates new evidence showing that a crucial national health service can adapt following a severe shock to the health system to maintain patient outcomes. Routine data can enable studies to generate useful evidence to inform future pandemic preparedness plans for core health services. Governments must ensure that appropriate legislation and capacity for curating the required data are in place prior to any future pandemic. This element of pandemic preparedness is essential for researchers to generate timely accurate assessment of how core health services are reacting to a health crises, which can help adapt guidance for service provision to reduce disruption and help maintain patient outcomes.

## Supplemental material

Supplemental Material - Assessing the resilience of a key health service: The response of acute surgical provision in England to the disruption of the COVID-19 pandemic Supplemental Material for Assessing the resilience of a key health service: The response of acute surgical provision in England to the disruption of the COVID-19 pandemic by Andrew Hutchings, Orlagh Carroll, Geoff Bellingan, David Cromwell, S Ramani Moonesinghe, Susan J Moug, Neil Smart, Ravinder Vohra, Robert J Hinchliffe and Richard Grieve in Journal of Health Services Research & Policy

## Data Availability

Data are provided under a data sharing agreement (reference DARS-NIC-583534-X7S2N) with NHS Digital (now NHS England) that does not allow external sharing of data. Data will be retained until expiry of the data sharing agreement. Queries can be sent to the corresponding author.*

## References

[1] Zhong L Lopez D Pei S , et al. Healthcare system resilience and adaptability to pandemic disruptions in the United States. Nat Med 2024; 30: 2311–2319. 10.1038/s41591-024-03103-6 38956198

[2] Kruk ME Myers M Varpilah ST , et al. What is a resilient health system? Lessons from ebola. Lancet 2015; 385: 1910–1912. 10.1016/S0140-6736(15)60755-3 25987159

[3] Fridell M Edwin S von Schreeb J , et al. Health system resilience: what are we talking about? A scoping review mapping characteristics and keywords. Int J Health Pol Manag 2019; 9: 6–16. 10.15171/ijhpm.2019.71 PMC6943300 31902190

[4] Witter S Thomas S Topp SM , et al. Health system resilience: a critical review and reconceptualisation. Lancet Global Health 2023; 11: e1454–e1458. 10.1016/S2214-109X(23)00279-6 37591591

[5] UK government response to the COVID-19 inquiry module 1 report. https://www.gov.uk/government/publications/uk-government-response-to-the-covid-19-inquiry-module-1-report/uk-government-response-to-the-covid-19-inquiry-module-1-report-html [Accessed February 9, 2025]

[6] Ledesma JR Chrysanthopoulou SA Lurie MN , et al. Health system resilience during the COVID-19 pandemic: a comparative analysis of disruptions in care from 32 countries. Health Serv Res 2024; 59: e14382. 10.1111/1475-6773.14382 PMC11622287 39295092

[7] Arsenault C Gage A Kim MK , et al. COVID-19 and resilience of healthcare systems in ten countries. Nat Med 2022; 28: 1314–1324. 10.1038/s41591-022-01750-1 PMC9205770 35288697

[8] COVIDSurg Collaborative . Mortality and pulmonary complications in patients undergoing surgery with perioperative SARS-CoV-2 infection: an international cohort study. Lancet 2020; 396: 27–38. 10.1016/S0140-6736(20)31182-X PMC7259900 32479829

[9] Hutchings A Moonesinghe R Moler ZS , et al. Impact of the first wave of COVID-19 on outcomes following emergency admissions for common acute surgical conditions: analysis of a national database in England. Br J Surg 2022; 109: 984–994. 10.1093/bjs/znac233 PMC9384585 35891605

[10] Royal College of Surgeons of England . Protecting surgery through a second wave. The Royal College of Surgeons of England. https://www.rcseng.ac.uk/coronavirus/protecting-surgery-through-a-second-wave/ Accessed 08/02/2025.

[11] Pople D Monk EJM Evans S , et al. Burden of SARS-CoV-2 infection in healthcare workers during second wave in England and impact of vaccines: prospective multicentre cohort study (SIREN) and mathematical model. BMJ 2022; 378: e070379. 10.1136/bmj-2022-070379 PMC9295077 35858689

[12] ICNARC report on COVID-19 in critical care: England . Wales and Northern Ireland 2021, Available from. https://www.icnarc.org/Our-Audit/Audits/Cmp/Reports Accessed 08/02/2025.

[13] Beamish AJ Brown C Abdelrahman T , et al. International surgical guidance for COVID-19: validation using an international Delphi process - cross-sectional study. Int J Surg 2020; 79: 309–316. 10.1016/j.ijsu.2020.06.015 PMC7282765 32531308

[14] El Boghdady M Ewalds-Kvist BM . Laparoscopic surgery and the debate on its safety during COVID-19 pandemic: a systematic review of recommendations. Surgeon 2021; 19: e29–e39. 10.1016/j.surge.2020.07.005 PMC7418789 32855070

[15] Palmer B . Chart of the week: the rise, fall and rise again of NHS staff sickness absences. Nuffield Trust. https://www.nuffieldtrust.org.uk/resource/chart-of-the-week-the-rise-fall-and-rise-again-of-nhs-staff-sickness-absences Accessed 08/02/2025.

[16] Grieve R Hutchings A Moler-Zapata S , et al. Effectiveness and cost-effectiveness of emergency surgery for adult emergency hospital admissions with common acute gastrointestinal conditions: the ESORT study. Health Soc Care Del Res 2023; 11: 1.

[17] Department of Health & Social Care and Office for National Statistics . Direct and indirect health impacts of COVID-19 in England: emerging omicron impacts. https://www.gov.uk/government/publications/direct-and-indirect-health-impacts-of-covid-19-in-england-emerging-omicron-impacts/direct-and-indirect-health-impacts-of-covid-19-in-england-emerging-omicron-impacts Accessed 08/02/2025.

[18] Armitage JN van der Meulen JH Royal College of Surgeons Co-morbidity Consensus Group . Identifying co-morbidity in surgical patients using administrative data with the royal college of surgeons charlson score. Br J Surg 2010; 97: 772–781. 10.1002/bjs.6930 20306528

[19] Jauhari Y Gannon MR Dodwell D , et al. Construction of the secondary care administrative records frailty (SCARF) index and validation on older women with operable invasive breast cancer in England and Wales: a cohort study. BMJ Open 2020; 10: e035395. 10.1136/bmjopen-2019-035395 PMC7223146 32376755

[20] Royston P Sauerbrei W . Multivariable Model-building: a pragmatic approach to regression analysis based on fractional polynomials for modelling continuous variables. John Wiley & Sons, 2008.

[21] Duclos A Cordier Q Polazzi S , et al. Excess mortality among non-COVID-19 surgical patients attributable to the exposure of French intensive and intermediate care units to the pandemic. Intensive Care Med 2023; 49: 313–323. 10.1007/s00134-023-07000-3 PMC9959950 36840798

[22] Elliott D Ochieng C Jepson M , et al. 'Overnight, things changed. Suddenly, we were in it': a qualitative study exploring how surgical teams mitigated risks of COVID-19. BMJ Open 2021; 11: e046662. 10.1136/bmjopen-2020-046662 PMC8210660 34135048

[23] Mouffak S Shubbar Q Saleh E , et al. Recent advances in management of COVID-19: a review. Biomed Pharmacother 2021; 143: 112107. 10.1016/j.biopha.2021.112107 PMC8390390 34488083

[24] Wilcox ME Rowan KM Harrison DA , et al. Does unprecedented ICU capacity strain, as experienced during the COVID-19 pandemic, impact patient outcome? Crit Care Med 2022; 50: e548–e556. 10.1097/CCM.0000000000005464 PMC9112508 35170537

[25] Surgical Royal Colleges of the United Kingdom and Ireland . Guidance for surgeons working during the COVID-19 pandemic from the surgical royal colleges of the United Kingdom and Ireland. https://www.rcseng.ac.uk/coronavirus/joint-guidance-for-surgeons-v1/ Accessed 09/02/2025.

[26] Doyle J Smith EMJS Gough CJR , et al. Mobilising a workforce to combat COVID-19: an account, reflections, and lessons learned. J Intensive Care Soc 2022; 23: 177–182. 10.1177/1751143720971540 PMC9125451 35615227

[27] Lo Sardo DR Thurner S Sorger J , et al. Quantification of the resilience of primary care networks by stress testing the health care system. Proc Natl Acad Sci USA 2019; 26(116): 23930–23935. 10.1073/pnas.1904826116 PMC6883827 31712415

[28] CODA Collaborative Flum DR Davidson GH , et al. A randomized trial comparing antibiotics with appendectomy for appendicitis. N Engl J Med 2020; 383: 1907–1919. 10.1056/NEJMoa2014320 33017106

[29] O’Leary DP Walsh SM Bolger J , et al. A randomized clinical trial evaluating the efficacy and quality of life of antibiotic-only treatment of acute uncomplicated appendicitis. Ann Surg 2021; 274: 240–247. 10.1097/SLA.0000000000004785 33534226

[30] Hutchings A O’Neill S Lugo-Palacios D , et al. Effectiveness of emergency surgery for five common acute conditions: an instrumental variable analysis of a national routine database. Anaesthesia 2022; 77: 865–881. 10.1111/anae.15730 PMC9540551 35588540

[31] The Strategy Unit . The case for surgical hubs. The Royal College of Surgeons of England, 2022. https://www.rcseng.ac.uk/-/media/files/rcs/about-rcs/government-relations-consultation/rcs--surgical-hubs-report.pdf Accessed December 30th, 2025.

