# Supplementary material for: Assessing the resilience of a key health service: The response of acute surgical provision in England to the disruption of the COVID-19 pandemic
Source: J Health Serv Res Policy. 2026 Mar 5;31(4):214–23. doi: 10.1177/13558196261426970 (PMC13589110; doi:10.1177/13558196261426970)

Table S1: Reasons for exclusion by wave and comparator group for appendicitis

	Wave 1	Wave 1 comparator	Wave 2	Wave 3	Wave 2/3 comparator	Other	Total
Episode in an emergency admission with a relevant primary diagnosis: n	4488	17,306	11,698	11,907	33,627	133,917	212,943
Exclusion criteria							
No episode with a consultant surgeon: n (%)	153 (3.4)	461 (2.7)	409 (3.5)	409 (3.4)	878 (2.6)	3702 (2.8)	6012 (2.8)
No eligible diagnosis in the first two episodes: n (%)	28 (0.6)	91 (0.5)	61 (0.5)	73 (0.6)	192 (0.6)	770 (0.6)	1215 (0.6)
Not admitted through A&E or GP: n (%)	427 (9.5)	1346 (7.8)	1232 (10.5)	1313 (11.0)	2700 (8.0)	11,795 (8.8)	18,813 (8.8)
Clinical panel exclusion criteria†: n (%)	30 (0.7)	100 (0.6)	54 (0.5)	57 (0.5)	167 (0.5)	654 (0.5)	1062 (0.5)
Missing discharge data: n (%)	7 (0.2)	20 (0.1)	14 (0.1)	11 (0.1)	48 (0.1)	211 (0.2)	311 (0.1)
Other admission meeting inclusion criteria in previous year: n (%)	161 (3.6)	277 (1.6)	381 (3.3)	290 (2.4)	495 (1.5)	2812 (2.1)	4416 (2.1)
Emergency surgery (ES) prior to index episode: n (%)	44 (1.0)	159 (0.9)	99 (0.8)	121 (1.0)	270 (0.8)	1269 (0.9)	1962 (0.9)
ES in prior admission within 90 days: n (%)	2 (0.0)	6 (0.0)	1 (0.0)	0 (0.0)	7 (0.0)	38 (0.0)	54 (0.0)
All exclusions: n (%)	852 (19.0)	2460 (14.2)	2251 (19.2)	2274 (19.1)	4757 (14.1)	21,251 (15.9)	33,845 (15.9)
Final cohort	3636	14,846	9447	9633	28,870	112,666	179,098

Table S2: Reasons for exclusion by wave and comparator group for gallstone disease

	Wave 1	Wave 1 comparator	Wave 2	Wave 3	Wave 2/3 comparator	Other	Total
Episode in an emergency admission with a relevant primary diagnosis: n	4913	21,937	15,456	16,305	41,570	172,779	272,960
Exclusion criteria							
No episode with a consultant surgeon: n (%)	803 (16.3)	3294 (15.0)	2376 (15.4)	2534 (15.5)	6276 (15.1)	27,141 (15.7)	42,424 (15.5)
No eligible diagnosis in the first two episodes: n (%)	77 (1.6)	312 (1.4)	220 (1.4)	240 (1.5)	643 (1.5)	2668 (1.5)	4160 (1.5)
Not admitted through A&E or GP: n (%)	416 (8.5)	1660 (7.6)	1538 (10.0)	1935 (11.9)	2885 (6.9)	14,392 (8.3)	22,826 (8.4)
Clinical panel exclusion criteria†: n (%)	0 (0.0)	0 (0.0)	0 (0.0)	0 (0.0)	0 (0.0)	0 (0.0)	0 (0.0)
Missing discharge data: n (%)	1 (0.0)	23 (0.1)	4 (0.0)	5 (0.0)	63 (0.2)	174 (0.1)	270 (0.1)
Other admission meeting inclusion criteria in previous year: n (%)	548 (11.2)	2385 (10.9)	1830 (11.8)	1871 (11.5)	4309 (10.4)	19,642 (11.4)	30,585 (11.2)
Emergency surgery (ES) prior to index episode: n (%)	8 (0.2)	33 (0.2)	18 (0.1)	25 (0.2)	57 (0.1)	214 (0.1)	355 (0.1)
ES in prior admission within 90 days: n (%)	11 (0.2)	35 (0.2)	24 (0.2)	22 (0.1)	76 (0.2)	282 (0.2)	450 (0.2)
All exclusions: n (%)	1864 (37.9)	7742 (35.3)	6010 (38.9)	6632 (40.7)	14,309 (34.4)	64,513 (37.3)	101,070 (37.0)
Final cohort	3049	14,195	9446	9673	27,261	108,266	171,890

Table S3: Reasons for exclusion by wave and comparator group for diverticular disease

	Wave 1	Wave 1 comparator	Wave 2	Wave 3	Wave 2/3 comparator	Other	Total
Episode in an emergency admission with a relevant primary diagnosis: n	2779	13,291	8510	9542	24,917	106,281	165,320
Exclusion criteria							
No episode with a consultant surgeon: n (%)	409 (14.7)	1637 (12.3)	1103 (13.0)	1258 (13.2)	3144 (12.6)	13,609 (12.8)	21,160 (12.8)
No eligible diagnosis in the first two episodes: n (%)	42 (1.5)	284 (2.1)	148 (1.7)	139 (1.5)	540 (2.2)	2057 (1.9)	3210 (1.9)
Not admitted through A&E or GP: n (%)	185 (6.7)	872 (6.6)	681 (8.0)	771 (8.1)	1631 (6.5)	7197 (6.8)	11,337 (6.9)
Clinical panel exclusion criteria†: n (%)	0 (0.0)	0 (0.0)	0 (0.0)	0 (0.0)	0 (0.0)	0 (0.0)	0 (0.0)
Missing discharge data: n (%)	3 (0.1)	10 (0.1)	1 (0.0)	6 (0.1)	20 (0.1)	112 (0.1)	152 (0.1)
Other admission meeting inclusion criteria in previous year: n (%)	295 (10.6)	1264 (9.5)	851 (10.0)	945 (9.9)	2398 (9.6)	10,174 (9.6)	15,927 (9.6)
Emergency surgery (ES) prior to index episode: n (%)	7 (0.3)	40 (0.3)	28 (0.3)	29 (0.3)	58 (0.2)	259 (0.2)	421 (0.3)
ES in prior admission within 90 days: n (%)	3 (0.1)	13 (0.1)	4 (0.0)	10 (0.1)	25 (0.1)	100 (0.1)	155 (0.1)
All exclusions: n (%)	944 (34.0)	4120 (31.0)	2816 (33.1)	3158 (33.1)	7816 (31.4)	33,508 (31.5)	52,362 (31.7)
Final cohort	1835	9171	5694	6384	17,101	72,773	112,958

Table S4: Reasons for exclusion by wave and comparator group for hernia

	Wave 1	Wave 1 comparator	Wave 2	Wave 3	Wave 2/3 comparator	Other	Total
Episode in an emergency admission with a relevant primary diagnosis: n	2184	9556	6383	7215	17,976	74,153	117,467
Exclusion criteria							
No episode with a consultant surgeon: n (%)	167 (7.6)	617 (6.5)	482 (7.6)	535 (7.4)	1201 (6.7)	5289 (7.1)	8291 (7.1)
No eligible diagnosis in the first two episodes: n (%)	18 (0.8)	126 (1.3)	55 (0.9)	62 (0.9)	218 (1.2)	713 (1.0)	1192 (1.0)
Not admitted through A&E or GP: n (%)	195 (8.9)	829 (8.7)	715 (11.2)	881 (12.2)	1467 (8.2)	7183 (9.7)	11,270 (9.6)
Clinical panel exclusion criteria†: n (%)	113 (5.2)	521 (5.5)	324 (5.1)	383 (5.3)	972 (5.4)	3804 (5.1)	6117 (5.2)
Missing discharge data: n (%)	2 (0.1)	7 (0.1)	3 (0.0)	2 (0.0)	24 (0.1)	70 (0.1)	108 (0.1)
Other admission meeting inclusion criteria in previous year: n (%)	166 (7.6)	598 (6.3)	509 (8.0)	488 (6.8)	1078 (6.0)	5141 (6.9)	7980 (6.8)
Emergency surgery (ES) prior to index episode: n (%)	12 (0.5)	48 (0.5)	44 (0.7)	54 (0.7)	111 (0.6)	440 (0.6)	709 (0.6)
ES in prior admission within 90 days: n (%)	12 (0.5)	51 (0.5)	23 (0.4)	21 (0.3)	103 (0.6)	361 (0.5)	571 (0.5)
All exclusions: n (%)	685 (31.4)	2797 (29.3)	2155 (33.8)	2426 (33.6)	5174 (28.8)	23001 (31.0)	36,238 (30.8)
Final cohort	1499	6759	4228	4789	12,802	51152	81,229

Table S5: Reasons for exclusion by wave and comparator group for intestinal obstruction

	Wave 1	Wave 1 comparator	Wave 2	Wave 3	Wave 2/3 comparator	Other	Total
Episode in an emergency admission with a relevant primary diagnosis: n	4546	15,214	10,594	11,452	29,887	117,254	188,947
Exclusion criteria							
No episode with a consultant surgeon: n (%)	633 (13.9)	2080 (13.7)	1451 (13.7)	1493 (13.0)	4053 (13.6)	15,788 (13.5)	25,498 (13.5)
No eligible diagnosis in the first two episodes: n (%)	78 (1.7)	449 (3.0)	249 (2.4)	246 (2.1)	881 (2.9)	3206 (2.7)	5109 (2.7)
Not admitted through A&E or GP: n (%)	309 (6.8)	925 (6.1)	625 (5.9)	705 (6.2)	1629 (5.5)	6615 (5.6)	10,808 (5.7)
Clinical panel exclusion criteria†: n (%)	264 (5.8)	734 (4.8)	590 (5.6)	674 (5.9)	1411 (4.7)	5975 (5.1)	9648 (5.1)
Missing discharge data: n (%)	4 (0.1)	27 (0.2)	13 (0.1)	12 (0.1)	64 (0.2)	219 (0.2)	339 (0.2)
Other admission meeting inclusion criteria in previous year: n (%)	788 (17.3)	2552 (16.8)	1714 (16.2)	1950 (17.0)	4905 (16.4)	19,414 (16.6)	31,323 (16.6)
Emergency surgery (ES) prior to index episode: n (%)	20 (0.4)	71 (0.5)	68 (0.6)	61 (0.5)	133 (0.4)	594 (0.5)	947 (0.5)
ES in prior admission within 90 days: n (%)	66 (1.5)	269 (1.8)	139 (1.3)	162 (1.4)	517 (1.7)	1930 (1.6)	3083 (1.6)
All exclusions: n (%)	2162 (47.6)	7107 (46.7)	4849 (45.8)	5303 (46.3)	13,593 (45.5)	53,741 (45.8)	86,755 (45.9)
Final cohort	2384	8107	5745	6149	16,294	63,513	102,192

Figure S1: The impact of subsequent Covid waves compared to wave 1

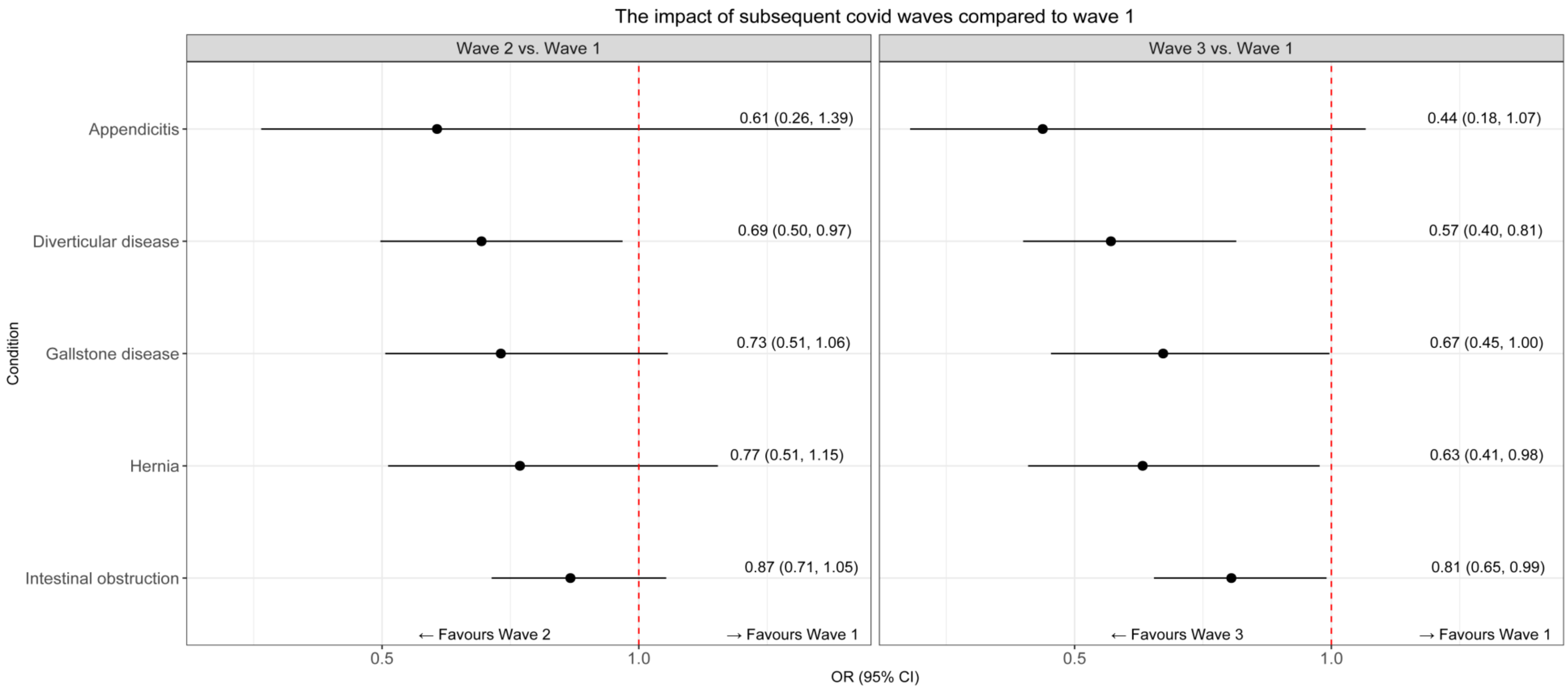

Figure S2: The impact of Covid during waves 1, 2 and 3 when excluding patients diagnosed with Covid at admission

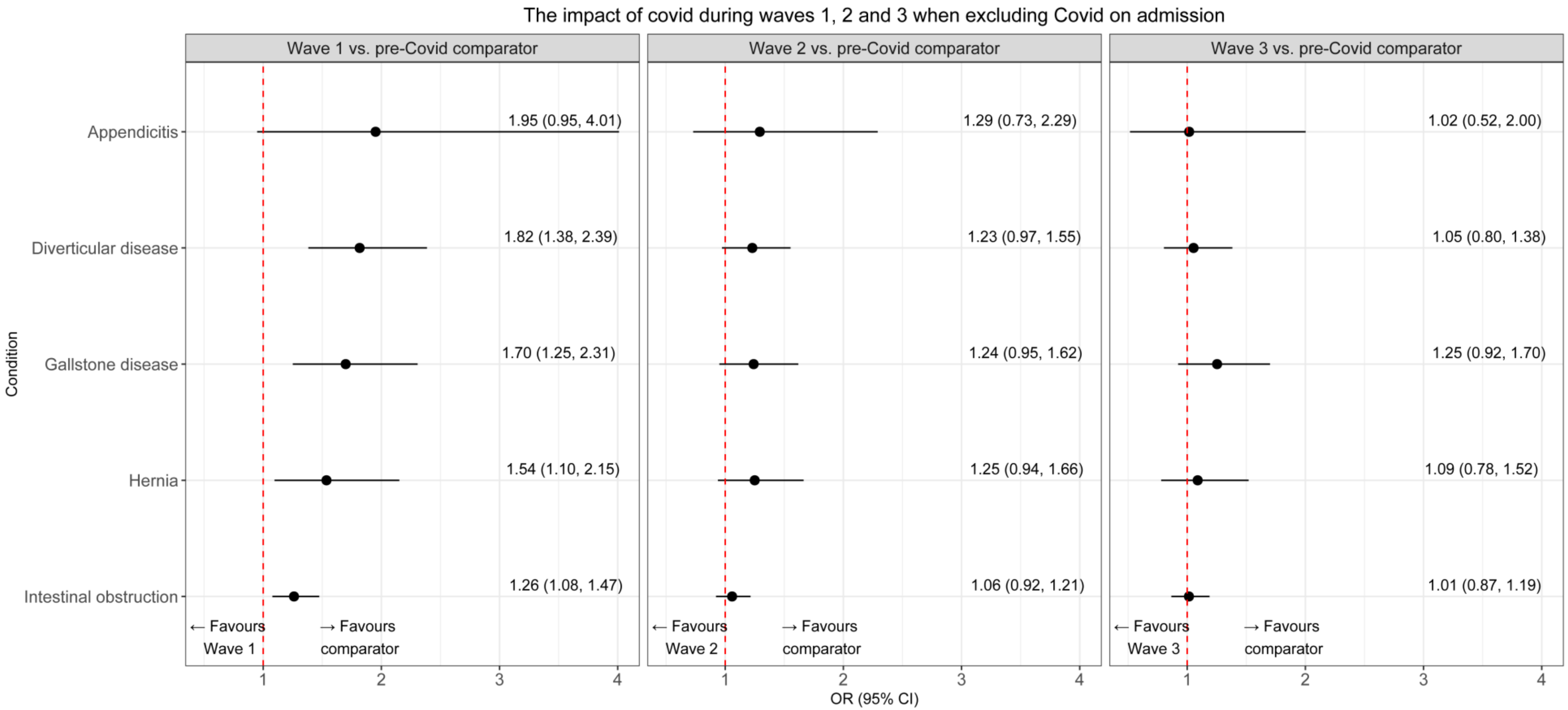

Figure S3: The impact of Covid during waves 1, 2 and 3 when excluding patients diagnosed with Covid at admission and up to 90 days

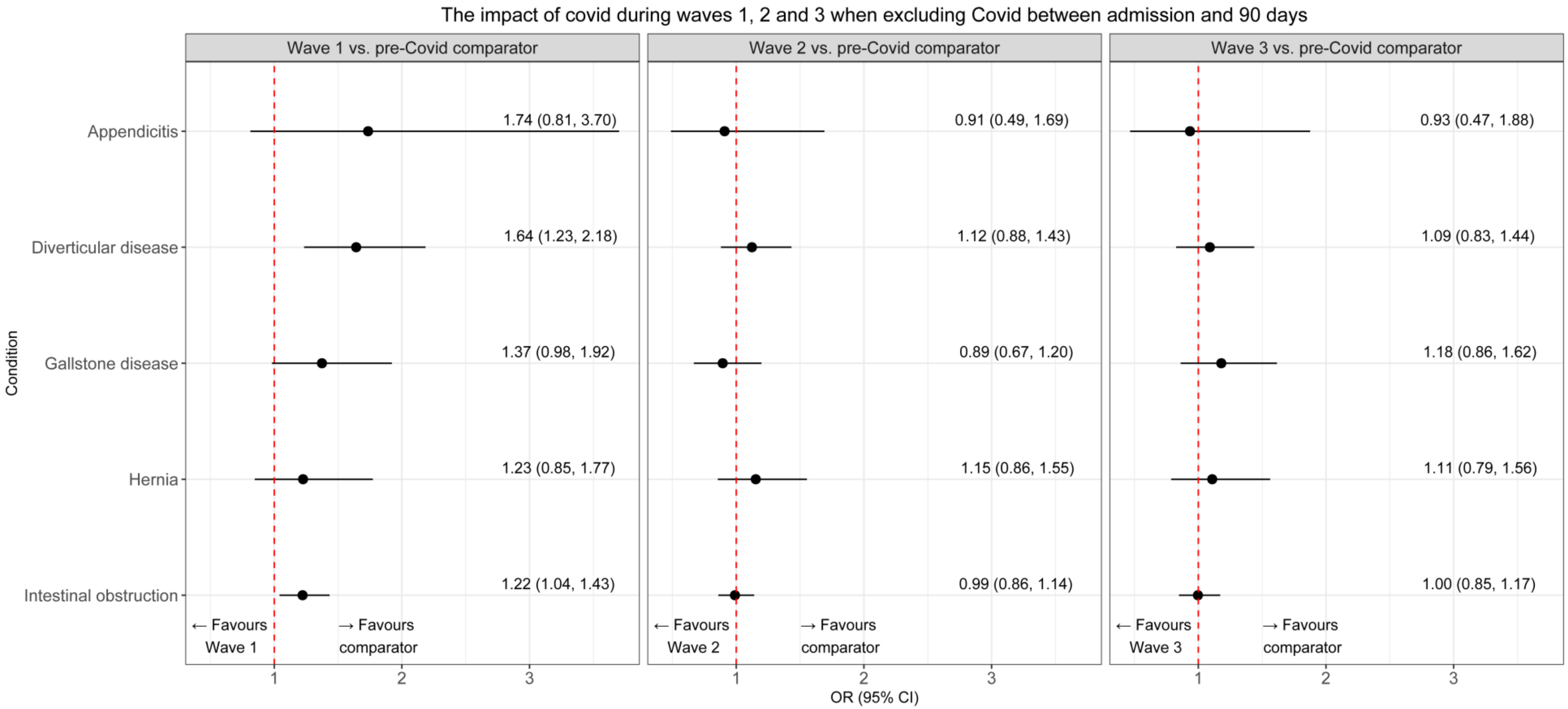

Supplement: Supplementary file 1 [file sj-pdf-1-hsr-10.1177_13558196261426970.pdf]
